# Transient Serotonin Syndrome by Concurrent Use of Electroconvulsive Therapy and Selective Serotonin Reuptake Inhibitor: A Case Report and Review of the Literature

**DOI:** 10.1155/2012/215214

**Published:** 2012-11-20

**Authors:** Nagahisa Okamoto, Kota Sakamoto, Maki Yamada

**Affiliations:** ^1^Department of Psychiatry, Sapporo Suzuki Hospital, 1-1-27 Sinkotoni 3jo, Kitaku, Sapporo City, Hokkaido 001-0903, Japan; ^2^Department of Psychiatry, National Center Hospital of Neurology and Psychiatry, 4-1-1 Ogawa Higashi, Kodaira City, Tokyo 187-8551, Japan

## Abstract

The serotonin syndrome, which is characterized by psychiatric, autonomic nervous and neurological symptoms, is considered to be caused by excessive stimulation of the 5-HT1A and 5-HT2 receptors in the gray matter and spinal cord of the central nervous system, after the start of dosing or increase of the dose of a serotoninergic drug. There have been hardly any reports of induction of serotonin syndrome by electroconvulsive therapy (ECT) in combination with antidepressant. We present the case of a female patient with major depressive disorder (MDD) who developed transient serotonin syndrome soon after the first session of ECT in combination with paroxetine. Paroxetine was discontinued, and her psychiatric, autonomic nervous and neurological symptoms were gradually relieved and disappeared within 2 days. We performed the second ECT session 5 days after the initial session and performed 12 sessions of ECT without any changes in the procedure of ECT and anesthesia, but no symptoms of SS were observed. Finally, her MDD remitted. ECT might cause transiently increased blood-brain barrier (BBB) permeability and enhance the transmissivity of the antidepressant in BBB. Therefore, it is necessary to pay attention to rare side effect of serotonin syndrome by ECT in combination with antidepressant.

## 1. Introduction

The serotonin syndrome, which is characterized by psychiatric, autonomic nervous and neurological symptoms, is considered to be caused by excessive stimulation of the 5-HT1A and 5-HT2 receptors in the gray matter and spinal cord of the central nervous system after the start of dosing or increase of the dose of a serotoninergic drug [1]. There have been hardly any reports of induction of serotonin syndrome by electroconvulsive therapy (ECT) in combination with antidepressant. We present the case of a female patient with major depressive disorder (MDD) who developed transient serotonin syndrome soon after the first session of ECT in combination with paroxetine. 

## 2. Case Presentation

Mrs. T was a 67-year-old woman who had no past history of physical disease and had no psychiatric family history. In June 2008, she experienced depressed mood, decreased energy, anxiety, agitation, and insomnia, and she visited a psychiatric clinic. She was diagnosed with MDD. She took milnacipran (30 mg/day), but her depressive symptoms became more serious gradually without a change of the medication around December 2008. She completely lost interest in her leisure activity. In April 2009, she developed physical symptoms such as a respiratory discomfort. She was examined in many physical hospitals, but no abnormal results were found. She developed hypochondriacal delusions and delusions of poverty gradually. Her appetite decreased, and her body weight was decreased by 12 kg in a year. 

She was admitted to a regional psychiatric hospital in May. She had severe agitation and complained, “I cannot breathe.” It was difficult for her to keep still, and she wandered about the hospital ward. She was treated sequentially with fluvoxamine (100 mg/day), amitriptyline (30 mg/day), sulpiride (50 mg/day), and augmentation olanzapine (10 mg/day), but it was impossible to use adequate doses because of her strong apprehension in regard to psychotropics. Finally, the neuroleptics and antidepressants were discontinued, and minor tranquilizers were prescribed, but her agitation did not improve. In October, paroxetine monotherapy (40 mg/day) was started, and she was transferred to us in November 2009. 

Severe anxiety, agitation, depressed mood, decreased energy, loss of appetite, decreased body weight, insomnia, hypochondriacal delusions, and delusions of poverty were noted, and she fulfilled the DSM-IV diagnostic criteria for MDD with psychotic features. Total score on the 17-item Hamilton Rating Scale for Depression (HRSD) was 31. The physical examination and neurological examination revealed only low body mass index of 16.6. The blood examination revealed only anemia (hemoglobin 11.4 g/dL). An electrocardiogram, electroencephalogram, and brain MRI revealed no abnormalities. 

ECT was done because she had severe depressive symptoms with psychotic feature. Anesthesia consisted of atropine sulfate (0.25 mg) intravenously with propofol (0.9 mg/kg) by intravenously bolus. Succinylcholine (0.9 mg/kg) was given intravenously as a muscle relaxant after induction of anesthesia. Thymatron IV system was used, and bifrontal brief pulse ECT was performed twice a week. The seizure threshold was determined by the half-age method. Paroxetine (40 mg/day), zolpidem (10 mg/day), and bromazepam (4 mg/day), those she had been taking at the previous hospital, were continued. 

Confusion persisted soon after awakening from anesthesia at the initial ECT session. 30 minutes later, she failed to respond to our calling and fell into a substupor. Concurrently she developed autonomic symptoms, including pyrexia (38.6°C), tachycardia (160/min), elevated blood pressure (160/70 mmHg), and severe hyperhidrosis, and neurological symptoms, including severe generalized rigidity, palpebral and labial myoclonus, tremors of all limbs, and generalized hyperreflexia. Her total score on the Serotonin Syndrome Scale (SSS) [2, 3] was 20, and she was diagnosed with the serotonin syndrome. Except for leukocytosis (12,250/*μ*L) and anemia (hemoglobin 9.9 g/dL), the blood examination revealed no abnormalities, including serum creatinine, blood urea nitrogen, creatinine phosphokinase, or electrolytes. The electroencephalogram revealed no paroxysmal abnormalities. In the same day, all oral medication including paroxetine was discontinued. Fluid administration was started under general management. Flunitrazepam (2 mg) was administered intravenously, and the neurological symptoms and autonomic symptoms were alleviated, but the benefit lasted only about 2 hr. The symptoms were gradually alleviated subsequently, and 24 hr later all vital signs except the pyrexia became normal. 48 hours later, the symptoms of serotonin syndrome including pyrexia and substupor resolved completely.

**Figure 1:**
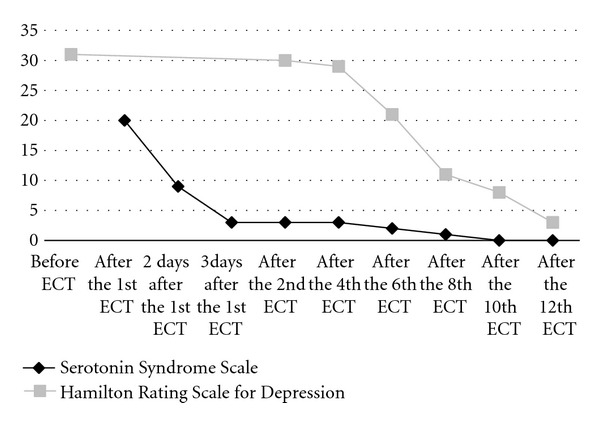
Time-course of changes in total score on Serotonin Syndrome Scale and Hamilton Rating Scale for Depression.

We continued the discontinuation of paroxetine, and performed the second ECT session 5 days after the initial session. We performed 12 sessions of ECT without any changes in the procedure of ECT and anesthesia, but no symptoms of serotonin syndrome were observed. Finally, her MDD remitted. The time-courses of the total scores on SSS and HRSD are shown (Figure 1).

## 3. Discussion

In our case, various neurological symptoms, autonomic symptoms, and mental status changes were observed. Because those symptoms were observed after only the initial ECT session with concurrent use of paroxetine, it was hard to explain those symptoms by induction of malignant hyperthermia induced by succinylcholine. Since 8 of 10 items of Sternbach's diagnostic criteria [4] were met, the diagnosis of serotonin syndrome was valid. 

The onset of SS generally occurs within 24 hr after the start of serotonergic drugs or increase in their dosage [1]. But there have been hardly any reports of serotonin syndrome induced by ECT. There have been only two case reports. In one case, serotonin syndrome was induced by ECT in combination with lithium and mirtazapine [5]. In another case, myoclonus like serotonin syndrome was induced by ECT monotherapy [6]. 

 Recently, a study suggested that ECT might cause a transiently increased blood-brain barrier (BBB) permeability in human [7]. In the study, plasma concentrations of amyloid *β* were measured in 13 patient before ECT, within 30 minutes after 2, and 24 hours after ECT treatment. They reported a significant increase of the plasma concentrations of amyloid *β* within 30 minutes after the ECT, followed by the normalization of the peptides concentrations 2 hours after the ECT. In this way, ECT might cause a transiently increased BBB permeability and enhance the transmissivity of the antidepressant in BBB. Moreover, it is known that serotonin reuptake inhibitor itself increases the efficacy of 5-HT neurons by desensitizing 5-HT autoreceptors located on serotonin nerve terminals and electroconvulsive shock treatment sensitize postsynaptic neurons to serotonin [8]. Therefore, serotonin syndrome might be induced by ECT in combination with antidepressants through two mechanisms. One mechanism is that the serotonin reuptake inhibitor itself and ECT itself enhance the activity of 5-HT neurons. Another mechanism is that ECT might cause transiently increased BBB permeability and increase the concentration of paroxetine in the brain to the toxic level.

Recently, a randomized placebo-controlled trial suggested that adding nortriptyline to ECT resulted in superior efficacy compared with adding placebo [9]. The concomitant administration of antidepressants with ECT probably increases acute antidepressant effect. Actually, ECT is often performed together with antidepressant that has the effect of the serotonin reuptake inhibition in our clinical setting. Therefore, our attention should be paid to serotonin syndrome with combination of ECT and antidepressant.

## 4. Conclusion

ECT might cause transiently increased BBB permeability and enhance the transmissivity of the antidepressant in BBB. Therefore, it is necessary to pay attention to rare side effect of serotonin syndrome by ECT in combination with antidepressant.
